# Supplementary material for: Oct4 mediates Müller glia reprogramming and cell cycle exit during retina regeneration in zebrafish
Source: Life Sci Alliance. 2019 Oct 8;2(5):e201900548. doi: 10.26508/lsa.201900548 (PMC6784428; doi:10.26508/lsa.201900548)
Supplement: Supplementary file 1 [file LSA-2019-00548_TableS1.docx]

| **Primers name** | **Ensembl ID** | **Sequence (5’-3’)** |
| --- | --- | --- |
| **Cloning primers** | | |
| ascl1a FL Fwd | \|  \| [ENSDART00000056005.5](http://asia.ensembl.org/Danio_rerio/Transcript/Summary?db=core;g=ENSDARG00000038386;r=4:17417111-17419193;t=ENSDART00000056005) \| \| --- \| --- \| | ATGGACATCACCGCCAAGATGGAAATAAGCG |
| ascl1a FL Rev |  | TCAAAACCAGTTGGTGAAGTCCAGGAGCTC |
| EcoR1-oct4-CDS-F | [ENSDART00000065817.4](http://asia.ensembl.org/Danio_rerio/Transcript/Summary?db=core;g=ENSDARG00000044774;r=21:13685853-13690712;t=ENSDART00000065817) | TACAGTCGAATTCCATGACGGAGAGAGCGCAGAG |
| Xho1-oct4-CDS-R |  | CACTAGTCTCGAGTTAGCTGGTGAGATGACCCACCAAAC |
| EcoR1-hdac1-CDS-F | [ENSDART00000186428.1](http://asia.ensembl.org/Danio_rerio/Transcript/Summary?db=core;g=ENSDARG00000015427;r=19:29808471-29821601;t=ENSDART00000186428) | cagtacgaattccATGGCGCTGAGTTCTCAAGGAAC |
| Xho1-hdac1-CDS-R |  | CACTAGTctcgagTCACACTGTTTTTAATTCTTCTTTTGGCC |
| BamH1-EGFP-F |  | GATGCTAGGATCCAGTCGCCACCATGGTGAGCAAG |
| EcoR1-EGFP-R |  | CAGGCTGAATTCTTGTACAGCTCGTCCATGC |
| lin28a FL BamI F | ENSDARG00000016999 | ATGCTGATGGATCCACCATGCCCCCGGCAAATCCGCATC |
| Lin28a FL XhoI R |  | ATGCTGATCTCGAGATCAGTGCTCTCTGGCAGTAAGGGAG |
| her4.1 FL Fwd | ENSDARG00000056732 | GAAACTCTACTGACAAACAAGCTG |
| her4.1 FL Rev |  | GATGTTGTCCATCTTCGTTTAGTGC |
| BamH1-tgfbi IS-F | [ENSDART00000105933.4](http://asia.ensembl.org/Danio_rerio/Transcript/Summary?db=core;g=ENSDARG00000071586;r=14:26511041-26536504;t=ENSDART00000105933) | ACTGACCGGATCCAGATGCTGGTCTTAATGATCACCTGGTC |
| Xho1-tgfbi IS-R |  | CAGCACTCTCGAGTGGTCACTCACAATTTTAGGAGGCAGTG |
| BamH1-snail1a CDS-F | [ENSDART00000130477.4](http://asia.ensembl.org/Danio_rerio/Transcript/Summary?db=core;g=ENSDARG00000056995;r=11:25252466-25257595;t=ENSDART00000130477) | ctagaatGGATCCATGCCTCGGTCTTTCCTGGTAAAG |
| Xba1-snail1a-CDS-R |  | atcgcataTCTAGACTATTGGACATTGGCCGTGGAGG |
| BamH1-snai2-CDS-F | [ENSDART00000058571.7](http://asia.ensembl.org/Danio_rerio/Transcript/Summary?db=core;g=ENSDARG00000040046;r=24:35387517-35393533;t=ENSDART00000058571) | ctagaatGGATCCATGCCTCGTTCATTCCTAGTAAAGAAG |
| Xba1-snai2-CDS-R |  | atcgcataTCTAGATCAGTGTGCGATGCAACAGC |
| BamH1-snai3-CDS-F | [ENSDART00000073549.6](http://asia.ensembl.org/Danio_rerio/Transcript/Summary?db=core;g=ENSDARG00000031243;r=7:55112922-55120300;t=ENSDART00000073549) | ctagaatGGATCCATGCCAAGGTCTTTCTTGGTGAAG |
| Xba1-snai3-CDS-R |  | atcgcataTCTAGACTAGGACATGGGACAGCACCCAG |
| BamH1-snai1b-CDS-F | [ENSDART00000067652.4](http://asia.ensembl.org/Danio_rerio/Transcript/Summary?db=core;g=ENSDARG00000046019;r=23:2502181-2513300;t=ENSDART00000067652) | ctagaatGGATCCATGCCACGCTCATTTCTTGTCAAG |
| Xba1-snai1b-CDS-R |  | atcgcataTCTAGACTAGAGCGCCGGACAGCAG |
| Cla1-zeb1a-CDS-F | [ENSDART00000005449.8](http://asia.ensembl.org/Danio_rerio/Transcript/Summary?db=core;g=ENSDARG00000016788;r=2:43807230-43852207;t=ENSDART00000005449) | atcacgATCGATCAATGGCAACTTGTGCAGTGAG |
| Xba1Xho1-zeb1a-CDS-R |  | atcacgTCTAGACTCGAGTCAATCCTCAGTTTCCATCTCC |
| EcoR1-zeb2a-CDS-F | [ENSDART00000189994.1](http://asia.ensembl.org/Danio_rerio/Transcript/Summary?db=core;g=ENSDARG00000062338;r=9:25568839-25837749;t=ENSDART00000189994) | atcacgGAATTCAATGAAGCAGGAGATCATGG |
| Xba1-zeb2a-CDS-R |  | atcacgTCTAGAAGTAGTTAATCGGCATCCTCG |
| **ChIP primers** | | |
| Oct4 BS on ascl1a P-F | ENSDARG00000038386 | TCTATGTGGAGCAGAGTTATAAGGCTG |
| Oct4 BS on ascl1a P-R |  | ACCTAAAGGTGTTTCCCCAGATTACACTTG |
| Oct4 BS1 on oct4 P-F | ENSDARG00000044774 | TGCATAAGAGGGCAGGAATGACAAAATG |
| Oct4 Bs1 on oct4 P-R |  | ACACAGGTTTTGCTAATCAATCGGAGTTG |
| Oct4 BS2 on oct4 P-F |  | ATCGCTACTCTGGATCTGGAAGC |
| Oct4 BS2 on oct4 P-R |  | AGCGAACATTGAGTTTGAGATGTTTGG |
| Oct4 BS3,4 on oct P-F |  | TGTTGGACACCAATCAAACCAACAC |
| Oct4 BS3 on oct4 P-R |  | AGCATGTGTTACAACTCTCTGTCTG |
| Oct4 BS4 on oct4 P-R |  | AGAGCAGGGCACAAAGTAACACTTTTTG |
| Oct4 BS on hdac1 int1-F | ENSDARG00000015427 | AGCAGTCACTGACTTTCAAACTACTACG |
| Oct4 BS on hdac1 intr1-R |  | TGACTTTATGTAGATGCCCTCCCTAAAG |
| Oct4 BS on chd4a P-F | ENSDARG00000063535 | TGCATCTCAGTTCATTTGGTGCACAAC |
| Oct4 BS on chd4a P-R |  | TTGTGTTAGCCACTGAAGCTTTGCAG |
| Sox2 BS on cdh1 P-F | ENSDARG00000102750 | TGCATTAGTGGGATGTACAGTAGATG |
| Sox2 BS on cdh1 P-R |  | AAATGAGAGATTACACATCGGCTG |
| Ascl1a BS on cdh1-F |  | AGTTCACTGATTAGGGTACCTGTG |
| Ascl1a BS on cdh1-R |  | AGCCATTTGACTCGCTACAAGTC |
| Oct4 BS on zeb1a P-F | ENSDARG00000016788 | ATACAATCCATTTTGACCACTGAAGCATG |
| Oct4 BS on zeb1a P-R |  | AGCATTAAGCATTTGAAGAGTGTAGATGC |
| Oct4 BS on zeb2a P-F | ENSDARG00000062338 | TCTGATGGGCTTTGTTTAACACCTGG |
| Oct4 BS on zeb2a P-R |  | AACCCACCACCCACCTAAATGC |
| Oct4 BS on zeb2b intr1-F | ENSDARG00000078416 | TGTGAAGCCACAGTGACCACTG |
| Oct4 BS on zeb2b intr1-R |  | AGAATCACCAACCAGATCCAGTCTG |
| Oct4 BS on zeb1b intr1-F | ENSDARG00000013207 | TGCTACCTATGCAGCAGGCCAC |
| Oct4 BS on zeb1b intr1-R |  | ACGCTCCACCTACACACACTG |
| Oct4 BS on miR-200a/b P-F | ENSDARG00000081399 | TGTACTTAAGAGCAGCAATCTGC |
| Oct4 BS on miR-200a/b P-R |  | TCCTACTAGACCCATATACAAATCG |
| Oct4 BS miR-143/145 P-F | ENSDARG00000082513 | ACTTACGTGAACTAAGTATGAACG |
| Oct4 BS miR-143/145 P-R |  | TGGTGACACTTTACAATAAGGTTG |
| Oct4 BS on Her4.1-F | ENSDARG00000056732 | ACCTTTAATGAAGGTAAGAGCATGACTGC |
| Oct4 BS on Her4.1-R |  | ACCACACTGCTTTCATTTACTGAAAGTGC |
| Ascl1a BS1 on oct4 P-F | ENSDARG00000044774 | TGCATAAGAGGGCAGGAATGACAAAATG |
| Ascl1a Bs1 on oct4 P-R |  | ACACAGGTTTTGCTAATCAATCGGAGTTG |
| Ascl1a BS2 on oct4 P-F |  | ATCGCTACTCTGGATCTGGAAGC |
| Ascl1a BS2 on oct4 P-R |  | AGCGAACATTGAGTTTGAGATGTTTGG |
| Ascl1a BS3,4 on oct P-F |  | TGTTGGACACCAATCAAACCAACAC |
| Ascl1a BS3 on oct4 P-R |  | AGCATGTGTTACAACTCTCTGTCTG |
| Ascl1a BS4 on oct4 P-R |  | AGAGCAGGGCACAAAGTAACACTTTTTG |
| **qPCR Primers** | | |
| oct4-RT-F | \|  \| [ENSDART00000065817.4](http://asia.ensembl.org/Danio_rerio/Transcript/Summary?db=core;g=ENSDARG00000044774;r=21:13685853-13690712;t=ENSDART00000065817) \| \| --- \| --- \| | AGATAACGCACATATCCGATGATCTAGGCCTG |
| oct4-RT-R |  | TGCGGGTGAGCATGCATGAATTGAGACATTG |
| actin-RT-F | [ENSDART00000054987.7](http://asia.ensembl.org/Danio_rerio/Transcript/Summary?db=core;g=ENSDARG00000037746;r=1:8649099-8653385;t=ENSDART00000054987) | GCAGAAGGAGATCACATCCCTGGC |
| actin-RT-R |  | CATTGCCGTCACCTTCACCGTTC |
| sox2-RT-F | \|  \| [ENSDART00000104493.5](http://asia.ensembl.org/Danio_rerio/Transcript/Summary?db=core;g=ENSDARG00000070913;r=22:37347896-37349967;t=ENSDART00000104493) \| \| --- \| --- \| | GAAAAACAGCCCGGACCGCATCAAGAGACC |
| sox2-RT-R |  | GTCTTGGTTTTCCTCCGGGGTCTGTATTTG |
| snai1a-RT-F | [ENSDART00000130477.4](http://asia.ensembl.org/Danio_rerio/Transcript/Summary?db=core;g=ENSDARG00000056995;r=11:25251495-25257595;t=ENSDART00000130477) | AGCTGGAATGTCAGAACGACACTTC |
| snai1a-RT-R |  | GTCTGACGTCCGTCCTTCATCTTC |
| snai1b-RT-F | [ENSDART00000067652.4](http://asia.ensembl.org/Danio_rerio/Transcript/Summary?db=core;g=ENSDARG00000046019;r=23:2502181-2513300;t=ENSDART00000067652) | ACACTGCAGCCCACAAGACGGCATC |
| snai1b-RT-R |  | CAGGAGAACGGACGCTCGCCGGTG |
| snai2-RT-F | \| [ENSDART00000058571.7](http://asia.ensembl.org/Danio_rerio/Transcript/Summary?db=core;g=ENSDARG00000040046;r=24:35387517-35393533;t=ENSDART00000058571) \|  \| \| --- \| --- \| | AGCACGTATTCGGGACTCATGAAGC |
| snai2-RT-R |  | CAGGAAAACGGTTTCTCACCCGTG |
| snai3-RT-F | [ENSDART00000073549.6](http://asia.ensembl.org/Danio_rerio/Transcript/Summary?db=core;g=ENSDARG00000031243;r=7:55112922-55120300;t=ENSDART00000073549) | AGTCAGGAACACAGCGATGAGTG |
| snai3-RT-R |  | ACGTGAATGGTTTCTCACCTGTG |
| tgfb1a-RT-F | \|  \| [ENSDART00000060839.3](http://asia.ensembl.org/Danio_rerio/Transcript/Summary?db=core;g=ENSDARG00000041502;r=15:2776506-2803313;t=ENSDART00000060839) \| \| --- \| --- \| | AGACCTGCTGTATGCGCAAGCTTTAC |
| tgfb1a-RT-R |  | ACCATGTTGGACAATTGCTCCACCTTG |
| tgfb1b-RT-F | [ENSDART00000134907.2](http://asia.ensembl.org/Danio_rerio/Transcript/Summary?db=core;g=ENSDARG00000034895;r=21:21621042-21629828;t=ENSDART00000134907) | ACGGGAAACAGATGCTGTTTGTACTGAAAAG |
| tgfb1b-RT-R |  | TAGAACCTGAGGTACACAGCAGGGCTGAG |
| tgfb3-RT-F | \|  \| [ENSDART00000019766.9](http://asia.ensembl.org/Danio_rerio/Transcript/Summary?db=core;g=ENSDARG00000019367;r=17:52059020-52091999;t=ENSDART00000019766) \| \| --- \| --- \| | ACCTCCAGACGACGAAAGCGAGCTCTC |
| tgfb3-RT-R |  | AGCGTGTTGTACAGACTCAGCAGCGAG- |
| tgfb2-RT-F | \|  \| [ENSDART00000148927.2](http://asia.ensembl.org/Danio_rerio/Transcript/Summary?db=core;g=ENSDARG00000027087;r=16:2745006-2807527;t=ENSDART00000148927) \|  \| \| --- \| --- \| --- \| | TGGATACTGCTTTCTGCTCCAGGAATG |
| tgfb2-RT-R |  | TTCCAGATCCTGAGACACGCAGCAG |
| tgfbi-RT-F | [ENSDART00000105933.4](http://asia.ensembl.org/Danio_rerio/Transcript/Summary?db=core;g=ENSDARG00000071586;r=14:26511041-26536504;t=ENSDART00000105933) | CGCTGACCTCAACAAACTCATGAGAG |
| tgfbi-RT-R |  | TGGTCACTCACAATTTTAGGAGGCAG |
| smad7-RT-F | [ENSDART00000009740.7](http://asia.ensembl.org/Danio_rerio/Transcript/Summary?db=core;g=ENSDARG00000016858;r=21:3419459-3452683;t=ENSDART00000009740) | ATATTCAGCCCCTATGGGGTTTTCAGATTC |
| smad7-RT-R |  | AGCCGATTTTGGCCCGAACCATTTGCAC |
| tgfbr1b-RT-F | [ENSDART00000061973.7](http://asia.ensembl.org/Danio_rerio/Transcript/Summary?db=core;g=ENSDARG00000042259;r=24:119680-150447;t=ENSDART00000061973) | ACGGGAAACAGATGCTGTTTGTACTGAAAAG |
| tgfbr1b-RT-R |  | TAGAACCTGAGGTACACAGCAGGGCTGAG |
| mycb-RT-F | [ENSDART00000005143.6](http://asia.ensembl.org/Danio_rerio/Transcript/Summary?db=core;g=ENSDARG00000007241;r=2:32016256-32018371;t=ENSDART00000005143) | AGTAGTGACAGCGAATCCGATGACG |
| mycb-RT-R |  | ATGTGGCTCTCGAATTTAATCCGC |
| ascl1a-RT-F | \|  \| [ENSDART00000056005.5](http://asia.ensembl.org/Danio_rerio/Transcript/Summary?db=core;g=ENSDARG00000038386;r=4:17417111-17419193;t=ENSDART00000056005) \|  \| \| --- \| --- \| --- \| | ATCTCCCAAAACTACTCTAATGACATGAACTCTAT |
| ascl1a-RT-R |  | CAAGCGAGTGCTGATATTTTTAAGTTTCCTTTTAC |
| lin28a-RT-F | [ENSDART00000193355.1](http://asia.ensembl.org/Danio_rerio/Transcript/Summary?db=core;g=ENSDARG00000016999;r=19:15440841-15473157;t=ENSDART00000193355) | TAACGTGCGGATGGGCTTCGGATTTCTGTC |
| lin28a-RT-R |  | ATTGGGTCCTCCACAGTTGAAGCATCGATC |
| her4.1-RT-F | [ENSDART00000079274.4](http://asia.ensembl.org/Danio_rerio/Transcript/Summary?db=core;g=ENSDARG00000056732;r=23:21452557-21453614;t=ENSDART00000079274) | GCTGATATCCTGGAGATGACG |
| her4.1-RT-R |  | GACTGTGGGCTGGAGTGTGTT |
| hdac1-RT-F | [ENSDART00000186428.1](http://asia.ensembl.org/Danio_rerio/Transcript/Summary?db=core;g=ENSDARG00000015427;r=19:29808471-29821601;t=ENSDART00000186428) | GACAGCACCATTCCTAATGAGCTCC |
| hdac1-RT-R |  | TATCGTGAGCACGAATGGAGATGCG |
| dla-RT-F | [ENSDART00000126339.3](http://asia.ensembl.org/Danio_rerio/Transcript/Summary?db=core;g=ENSDARG00000010791;r=1:54013457-54026352;t=ENSDART00000126339) | GCGCAGGAAACGTCTGAAAAGTGAC |
| dla-RT-R |  | ATCCTGCAGGCCCATTACACCTCAG |
| dlb-RT-F | [ENSDART00000019259.9](http://asia.ensembl.org/Danio_rerio/Transcript/Summary?db=core;g=ENSDARG00000004232;r=5:36693859-36701760;t=ENSDART00000019259) | AAGAATGGCGGCAGTTGTAATGATTTG |
| dlb-RT-R |  | AGATCCACACATTCACCACCGTTG |
| dlc-RT-F | [ENSDART00000018514.8](http://asia.ensembl.org/Danio_rerio/Transcript/Summary?db=core;g=ENSDARG00000002336;r=15:20463448-20468302;t=ENSDART00000018514) | GAGCACCTCAAACACCAG |
| dlc-RT-R |  | CACCTCCTCCACCCATAA |
| dld-RT-F | [ENSDART00000099224.4](http://asia.ensembl.org/Danio_rerio/Transcript/Summary?db=core;g=ENSDARG00000020219;r=13:6075572-6081803;t=ENSDART00000099224) | AAATGGAGGAAGTTGCACTGATC |
| dld-RT-R |  | AAGATCGAGACACTGAGCATCATTC |
| il11a-RT-F | \| [ENSDART00000055160.8](http://asia.ensembl.org/Danio_rerio/Transcript/Summary?db=core;g=ENSDARG00000037859;r=16:12611362-12617155;t=ENSDART00000055160) \|  \| \| --- \| --- \| | CTCCTCATCGCTGCTTCTCTCG |
| il11a-RT-R |  | TTGCGAAGTCACTGGCTCTGC |
| il11b-RT-F | \|  \| [ENSDART00000081440.4](http://asia.ensembl.org/Danio_rerio/Transcript/Summary?db=core;g=ENSDARG00000058557;r=19:10429810-10432134;t=ENSDART00000081440) \| \| --- \| --- \| | GCTAACAGTGTCGCCTGACTCC |
| il11b-RT-R |  | CTGTAGTTCAGTGAGGGCAGGG |
| lepa-RT-F | [ENSDART00000126441.2](http://asia.ensembl.org/Danio_rerio/Transcript/Summary?db=core;g=ENSDARG00000091085;r=18:10689772-10690365;t=ENSDART00000126441) | TTTCCAGCTCTCCGCTCAACC |
| lepa-RT-R |  | CGGCGTATCTGGTCAACATGC |
| lepb-RT-F | [ENSDART00000133203.2](http://asia.ensembl.org/Danio_rerio/Transcript/Summary?db=core;g=ENSDARG00000045548;r=4:19031768-19033805;t=ENSDART00000133203) | CATTGCTCGAACCACCATCAGC |
| lepb-RT-R |  | TCTTTATGCACCGGGGTCTCG |
| lepr-RT-F | \|  \| [ENSDART00000079173.6](http://asia.ensembl.org/Danio_rerio/Transcript/Summary?db=core;g=ENSDARG00000070961;r=6:31184058-31233696;t=ENSDART00000079173) \| \| --- \| --- \| | CAGTACGAGCTGCAATTCAAGG |
| lepr-RT-R |  | TAAAATGCGCCAGAAGTCTGG |
| mmp9-RT-F | \| [ENSDART00000062845.5](http://asia.ensembl.org/Danio_rerio/Transcript/Summary?db=core;g=ENSDARG00000042816;r=8:24281512-24295947;t=ENSDART00000062845) \|  \| \| --- \| --- \| | GGAGAAAACTTCTGGAGACTTG |
| mmp9-RT-R |  | CACTGAAGAGAAACGGTTTCC |
| mmp2-RT-F | [ENSDART00000026712.8](http://asia.ensembl.org/Danio_rerio/Transcript/Summary?db=core;g=ENSDARG00000017676;r=7:35410050-35432901;t=ENSDART00000026712) | CTTAGCCTTAATGGTGATGGTC |
| mmp2-RT-R |  | GCTTACTGTGGGTCCAGGCAG |
| chd3-RT-F | [ENSDART00000188267.1](http://asia.ensembl.org/Danio_rerio/Transcript/Summary?db=core;g=ENSDARG00000021405;r=7:20673287-20758828;t=ENSDART00000188267) | TTCCTGGCAAGACGGTTCAAGTTG |
| chd3-RT-R |  | TCCTCCAACTGGTTTAGCACCTTG |
| chd4a-RT-F | \|  \| [ENSDART00000130062.3](http://asia.ensembl.org/Danio_rerio/Transcript/Summary?db=core;g=ENSDARG00000063535;r=19:5000885-5058908;t=ENSDART00000130062) \| \| --- \| --- \| | AGTTTCTTGCCCGGAGGTTCAAGTTG |
| chd4a-RT-R |  | TATGTAGGACGGCGTTGGCAGGTTTG |
| chd4b-RT-F | \|  \| [ENSDART00000184655.1](http://asia.ensembl.org/Danio_rerio/Transcript/Summary?db=core;g=ENSDARG00000025789;r=16:31761825-31791165;t=ENSDART00000184655) \| \| --- \| --- \| | TGGTGATTGAGGAGCAGCTCAGACGTG |
| chd4b-RT-R |  | AAGCTCCTCCAGTTGTTTGAGAACTTTG |
| ccna1-RT-F | [ENSDART00000002166.8](http://asia.ensembl.org/Danio_rerio/Transcript/Summary?db=core;g=ENSDARG00000043236;r=10:34905980-34916208;t=ENSDART00000002166)   \|  \|  \| \| --- \| --- \| | GAAGTACAAGAGCTCGAAATATC |
| ccna1-RT-R |  | GATCGCTTTATAATCGTGCAC |
| ccnb1-RT-F | [ENSDART00000063357.6](http://asia.ensembl.org/Danio_rerio/Transcript/Summary?db=core;g=ENSDARG00000051923;r=5:54711027-54714789;t=ENSDART00000063357) | GCCTTTCTAAGCATCTGGCTG |
| ccnb1-RT-R |  | CTTTTGCTCAACCCATGGCAG |
| ccnd1-RT-F | \|  \| [ENSDART00000163748.2](http://asia.ensembl.org/Danio_rerio/Transcript/Summary?db=core;g=ENSDARG00000101637;r=7:54668303-54679595;t=ENSDART00000163748) \|  \| \| --- \| --- \| --- \| | GTCATCAGAAGTGACCCTGACTG |
| ccnd1-RT-R |  | CAAAGCCCATACCCATCAGAAAC |
| ccne1-RT-F | \|  \| [ENSDART00000163991.3](http://asia.ensembl.org/Danio_rerio/Transcript/Summary?db=core;g=ENSDARG00000098622;r=7:46019780-46029879;t=ENSDART00000163991) \| \| --- \| --- \| | CCTACTTGGAATGGCTGGGAAAG |
| ccne1-RT-R |  | CTTATGCCAATGCTTGTGAATGC |
| miR-143-RT-F | \|  \| [ENSDART00000116314.2](http://asia.ensembl.org/Danio_rerio/Transcript/Summary?db=core;g=ENSDARG00000082513;r=14:38742313-38742411;t=ENSDART00000116314) \| \| --- \| --- \| | GTCGTCTGGCCCGCGGTGCAG |
| miR-143-RT-R |  | GACAGTGTTGTCCTCCCG AGC |
| miR-145-RT-F | \|  \| [ENSDART00000115736.4](http://asia.ensembl.org/Danio_rerio/Transcript/Summary?db=core;g=ENSDARG00000083100;r=14:38743941-38744053;t=ENSDART00000115736) \| \| --- \| --- \| | CTTCATCATTTCCTCATCCC |
| miR-145-RT-R |  | GTAGTCCACCCCCAACCCCAAG |
| miR-200a-RT-F | \|  \| [ENSDART00000117439.2](http://asia.ensembl.org/Danio_rerio/Transcript/Summary?db=core;g=ENSDARG00000081399;r=23:24072160-24072245;t=ENSDART00000117439) \| \| --- \| --- \| | GCACTTAGCAGCCATCTTACCGG |
| miR-200a-RT-R |  | TCACCCAACAAACATCGTTAC |
| miR-200b-RT-F | \|  \| [ENSDART00000115463.2](http://asia.ensembl.org/Danio_rerio/Transcript/Summary?db=core;g=ENSDARG00000081868;r=23:24072029-24072110;t=ENSDART00000115463) \| \| --- \| --- \| | GTAGTCGTCTCCATCTTACGAG |
| miR-200b-RT-R |  | GCAGCAATCATCATCATTACCAG |
| zeb1a-RT-F | \|  \| [ENSDART00000005449.8](http://asia.ensembl.org/Danio_rerio/Transcript/Summary?db=core;g=ENSDARG00000016788;r=2:43807230-43852207;t=ENSDART00000005449) \| \| --- \| --- \| | ACATGTCAATGGCATCAAGGAGG |
| zeb1a-RT-R |  | ATTCATGAGGCCTCTTTCCTGTG |
| zeb1b-RT-F | \|  \| [ENSDART00000047724.6](http://asia.ensembl.org/Danio_rerio/Transcript/Summary?db=core;g=ENSDARG00000013207;r=12:26750274-26851726;t=ENSDART00000047724) \|  \| \| --- \| --- \| --- \| | ACACAAGTATGAACACACAGGCAAG |
| zeb1b-RT-R |  | TGCTTATGTGTTCGGGCAGCTCC |
| zeb2a-RT-F | \|  \|  \|  \| [ENSDART00000127834.4](http://asia.ensembl.org/Danio_rerio/Transcript/Summary?db=core;g=ENSDARG00000062338;r=9:25568839-25837749;t=ENSDART00000127834) \| \| --- \| --- \| --- \| --- \| | AAGCCAGCATACCGGGTCTAAGG |
| zeb2a-RT-R |  | TGGTGAGGCCGCTTTCCACTTTC |
| zeb2b-RT-F | \|  \| [ENSDART00000090019.4](http://asia.ensembl.org/Danio_rerio/Transcript/Summary?db=core;g=ENSDARG00000078416;r=6:840434-957906;t=ENSDART00000090019) \| \| --- \| --- \| | TGGTCTAAACCCGTTCGCTGC |
| zeb2b-RT-R |  | CGTCAAGAAGCTCGCTCTGG |
| cdh1-RT-F | [ENSDART00000168890.2](http://asia.ensembl.org/Danio_rerio/Transcript/Summary?db=core;g=ENSDARG00000102750;r=7:53059299-53117237;t=ENSDART00000168890) | CTGTTTGAGCAATCCTCGTACAC |
| cdh1-RT-R |  | GTCTTCTCATAGTCCAGAGGCTTC |
